# Poly[bis­(μ_2_-pyrimidine-2-carboxyl­ato-κ^4^ *O*,*N*:*O*′,*N*′)calcium]

**DOI:** 10.1107/S1600536809025537

**Published:** 2009-07-08

**Authors:** Bing-Yu Zhang, Jing-Jing Nie, Duan-Jun Xu

**Affiliations:** a Department of Chemistry, Zhejiang University, People’s Republic of China

## Abstract

In the crystal structure of the title polymeric complex, [Ca(C_5_H_3_N_2_O_2_)_2_]_*n*_, the Ca^II^ cation has site symmetry 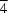 *m*2 and is *N*,*O*-chelated by four pyrimidine-2-carboxyl­ate anions in a square-anti­prismatic geometry. The planar pyrimidine-2-carboxyl­ate anion is located on a crystallographic special position, three C atoms have site symmetry 2*mm*, while the carboxyl O atom, the pyrimidine N atom and the other C atom have site symmetry *m*. Each pyrimidine-2-­carboxyl­ate anion bridges two Ca^II^ cations, forming polymeric sheets extending parallel to (001). π–π stacking exists between parallel pyrimidine rings [centroid–centroid distance = 3.6436 (6) Å] of adjacent polymeric sheets. Weak C—H⋯O hydrogen bonding is also observed between these sheets.

## Related literature

For general background, see: Deisenhofer & Michel (1989 ▶); Pan & Xu (2004 ▶); Li *et al.* (2005 ▶). For polymeric structures of metal complexes with the pyrimidine-2-carboxyl­ate ligand, see: Rodríguez-Diéguez *et al.* (2007 ▶, 2008 ▶); Zhang *et al.* (2008*a* ▶,*b* ▶); Sava *et al.* (2008 ▶). For mononuclear metal complexes of pyrimidine-2-carboxyl­ate, see: Antolić *et al.* (2000 ▶); Zhang *et al.* (2008 ▶); Xu *et al.* (2008 ▶). For Ca—N and Ca—O bond distances in *N*,*O*-chelated complexes, see: Starosta & Leciejewicz (2004 ▶).
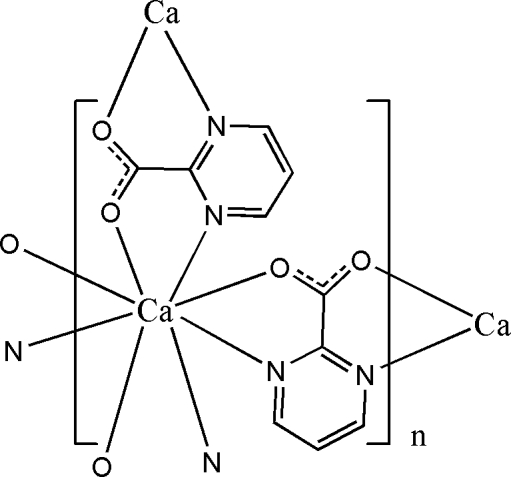


## Experimental

### 

#### Crystal data

[Ca(C_5_H_3_N_2_O_2_)_2_]
*M* *_r_* = 286.27
Tetragonal, 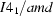

*a* = 6.5312 (12) Å
*c* = 25.734 (3) Å
*V* = 1097.7 (3) Å^3^
*Z* = 4
Mo *K*α radiation
μ = 0.59 mm^−1^
*T* = 294 K
0.22 × 0.20 × 0.14 mm

#### Data collection

Rigaku R-AXIS RAPID IP diffractometer
Absorption correction: multi-scan (*ABSCOR*; Higashi, 1995 ▶) *T*_min_ = 0.85, *T*_max_ = 0.92
3191 measured reflections
375 independent reflections
364 reflections with *I* > 2σ(*I*)
*R*_int_ = 0.016

#### Refinement

*R*[*F* ^2^ > 2σ(*F* ^2^)] = 0.025
*wR*(*F* ^2^) = 0.068
*S* = 1.13
375 reflections
34 parameters
H-atom parameters constrained
Δρ_max_ = 0.22 e Å^−3^
Δρ_min_ = −0.17 e Å^−3^

### 

Data collection: *PROCESS-AUTO* (Rigaku, 1998 ▶); cell refinement: *PROCESS-AUTO*; data reduction: *CrystalStructure* (Rigaku/MSC, 2002 ▶); program(s) used to solve structure: *SIR92* (Altomare *et al.*, 1993 ▶); program(s) used to refine structure: *SHELXL97* (Sheldrick, 2008 ▶); molecular graphics: *ORTEP-3 for Windows* (Farrugia, 1997 ▶); software used to prepare material for publication: *WinGX* (Farrugia, 1999 ▶).

## Supplementary Material

Crystal structure: contains datablocks I, global. DOI: 10.1107/S1600536809025537/hk2721sup1.cif

Structure factors: contains datablocks I. DOI: 10.1107/S1600536809025537/hk2721Isup2.hkl

Additional supplementary materials:  crystallographic information; 3D view; checkCIF report

## Figures and Tables

**Table 1:** Selected bond lengths (Å)

Ca—O1	2.3644 (11)
Ca—N1	2.6923 (13)

**Table 2:** Hydrogen-bond geometry (Å, °)

*D*—H⋯*A*	*D*—H	H⋯*A*	*D*⋯*A*	*D*—H⋯*A*
C3—H3⋯O1^i^	0.93	2.57	3.3689 (19)	144

Symmetry code: (i) 
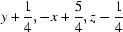
.

**Fig. 1.**
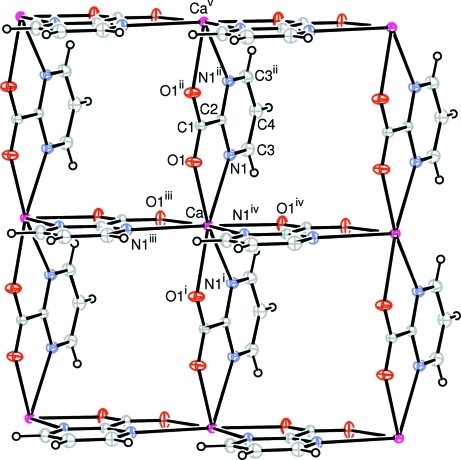
A part of polymeric structure of the title compound with 30% probability displacement ellipsoids for non-H atoms (arbitrary spheres for H atoms) [symmetry codes: (i) 1 - x, 3/2 - y, z; (ii) 1 - x, 1/2 - y, z; (iii) 5/4 - y, 1/4 + x, 3/4 - z; (iv) -1/4 + y, 1/4 + x, 3/4 - z; (v) x, -1 + y, z].

**Fig. 2.**
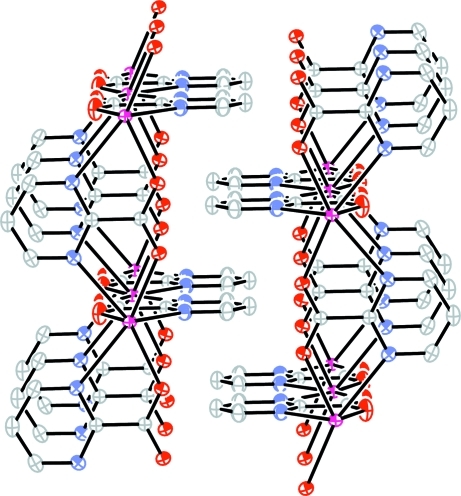
A diagram showing π-π stacking between parallel pyrimidine rings of adjacent polymeric sheets.

## supplementary crystallographic information

### Comment

As π-π stacking between aromatic rings is correlated with the electron transfer process in some biological systems (Deisenhofer & Michel, 1989), a series metal complexes incorporating the aromatic compound has been prepared in our laboratory to investigate the nature of π-π stacking (Li *et al.*, 2005; Pan & Xu, 2004). We report herein the crystal structure of the title compound of pyridinecarboxylate to show π-π stacking in the crystal structure.

A part of the polymeric structure of the title molecule is shown in Fig. 1. In the crystal structure, the Ca^II^ cation has site symmetry -4*m*2 and is N,*O*-chelated by four pyrimidinecarboxylate anions with the square-antiprism geometry. The Ca—N and Ca—O bond distances (Table 1) agree with those found in the N,*O*-chelated Ca^II^ complex (Starosta & Leciejewicz, 2004). The planar pyrimidinecarboxylate anion is located on the crystallographic special position, three C atoms have site symmetry 2 mm while the carboxyl O atom, the pirimidine N atom and the other C atom have site symmetry m. Each pyrimidinecarboxylate anion N,*O*-chelates two Ca^II^ cations (Antolić *et al.*, 2000; Zhang *et al.*, 2008; Xu *et al.*, 2008), forming the two-dimensional polymeric sheets, similar to those found in reported compounds (Rodríguez-Diéguez *et al.*, 2007, 2008; Zhang *et al.*, 2008*a*,b; Sava *et al.* 2008). π-π stacking [centroid-centroid distance = 3.6436 (6) Å] exists between parallel pyrimidine rings of adjacent polymeric sheets (Fig. 2). Weak C—H···O hydrogen bonding is also observed between polymeric sheets (Table 2).

### Experimental

2-Cyanopyrimidine (0.2 g, 2 mmol), NaOH (1.2 g, 30 mmol) and calcium chloride (0.1 g, 1 mmol) were dissolved in water (10 ml). The solution was refluxed for 3 h. After cooling to room temperature the solution was filtered. The single crystals were obtained from the filtrate after 5 d.

### Refinement

H atoms were placed in calculated positions with C—H = 0.93 Å and refined in riding mode with *U*_iso_(H) = 1.2*U*_eq_(C).

### Crystal data

**Table:**

[Ca(C_5_H_3_N_2_O_2_)_2_]	*D*_x_ = 1.732 Mg m^−^^3^
*M**_r_* = 286.27	Mo *K*α radiation, λ = 0.71073 Å
Tetragonal, *I*4_1_/*a**m**d*	Cell parameters from 1086 reflections
Hall symbol: -I 4bd 2	θ = 3.2–25.0°
*a* = 6.5312 (12) Å	µ = 0.59 mm^−^^1^
*c* = 25.734 (3) Å	*T* = 294 K
*V* = 1097.7 (3) Å^3^	Block, colorless
*Z* = 4	0.22 × 0.20 × 0.14 mm
*F*(000) = 584	

### Data collection

**Table:**

Rigaku R-AXIS RAPID IP diffractometer	375 independent reflections
Radiation source: fine-focus sealed tube	364 reflections with *I* > 2σ(*I*)
graphite	*R*_int_ = 0.016
ω scans	θ_max_ = 27.5°, θ_min_ = 3.2°
Absorption correction: multi-scan (*ABSCOR*; Higashi, 1995)	*h* = −8→8
*T*_min_ = 0.85, *T*_max_ = 0.92	*k* = −7→8
3191 measured reflections	*l* = −14→33

### Refinement

**Table:**

Refinement on *F*^2^	Secondary atom site location: difference Fourier map
Least-squares matrix: full	Hydrogen site location: inferred from neighbouring sites
*R*[*F*^2^ > 2σ(*F*^2^)] = 0.025	H-atom parameters constrained
*wR*(*F*^2^) = 0.068	*w* = 1/[σ^2^(*F*_o_^2^) + (0.0407*P*)^2^ + 0.7773*P*] where *P* = (*F*_o_^2^ + 2*F*_c_^2^)/3
*S* = 1.13	(Δ/σ)_max_ < 0.001
375 reflections	Δρ_max_ = 0.22 e Å^−^^3^
34 parameters	Δρ_min_ = −0.17 e Å^−^^3^
0 restraints	Extinction correction: *SHELXL97* (Sheldrick, 2008), Fc^*^=kFc[1+0.001xFc^2^λ^3^/sin(2θ)]^-1/4^
Primary atom site location: structure-invariant direct methods	Extinction coefficient: 0.071 (5)

### Special details

**Table:**

Geometry. All e.s.d.'s (except the e.s.d. in the dihedral angle between two l.s. planes) are estimated using the full covariance matrix. The cell e.s.d.'s are taken into account individually in the estimation of e.s.d.'s in distances, angles and torsion angles; correlations between e.s.d.'s in cell parameters are only used when they are defined by crystal symmetry. An approximate (isotropic) treatment of cell e.s.d.'s is used for estimating e.s.d.'s involving l.s. planes.
Refinement. Refinement of *F*^2^ against ALL reflections. The weighted *R*-factor *wR* and goodness of fit *S* are based on *F*^2^, conventional *R*-factors *R* are based on *F*, with *F* set to zero for negative *F*^2^. The threshold expression of *F*^2^ > σ(*F*^2^) is used only for calculating *R*-factors(gt) *etc*. and is not relevant to the choice of reflections for refinement. *R*-factors based on *F*^2^ are statistically about twice as large as those based on *F*, and *R*- factors based on ALL data will be even larger.

### Fractional atomic coordinates and isotropic or equivalent isotropic displacement parameters (Å^2^)

**Table:**

	*x*	*y*	*z*	*U*_iso_*/*U*_eq_	
Ca	0.5000	0.7500	0.3750	0.0164 (3)	
N1	0.5000	0.4327 (2)	0.30820 (5)	0.0226 (4)	
O1	0.5000	0.41994 (18)	0.41274 (4)	0.0292 (4)	
C1	0.5000	0.2500	0.39085 (8)	0.0197 (5)	
C2	0.5000	0.2500	0.33146 (8)	0.0188 (5)	
C3	0.5000	0.4306 (3)	0.25605 (6)	0.0299 (4)	
H3	0.5000	0.5542	0.2381	0.036*	
C4	0.5000	0.2500	0.22845 (10)	0.0319 (6)	
H4	0.5000	0.2500	0.1923	0.038*	

### Atomic displacement parameters (Å^2^)

**Table:**

	*U*^11^	*U*^22^	*U*^33^	*U*^12^	*U*^13^	*U*^23^
Ca	0.0152 (3)	0.0152 (3)	0.0189 (4)	0.000	0.000	0.000
N1	0.0254 (7)	0.0209 (7)	0.0215 (7)	0.000	0.000	0.0026 (5)
O1	0.0499 (8)	0.0169 (6)	0.0209 (6)	0.000	0.000	−0.0015 (4)
C1	0.0224 (10)	0.0181 (10)	0.0186 (10)	0.000	0.000	0.000
C2	0.0170 (9)	0.0201 (10)	0.0193 (10)	0.000	0.000	0.000
C3	0.0345 (9)	0.0326 (9)	0.0226 (8)	0.000	0.000	0.0072 (7)
C4	0.0337 (13)	0.0438 (15)	0.0184 (10)	0.000	0.000	0.000

### Geometric parameters (Å, °)

**Table:**

Ca—O1^i^	2.3644 (12)	N1—C3	1.342 (2)
Ca—O1^ii^	2.3644 (11)	O1—C1	1.2447 (15)
Ca—O1	2.3644 (11)	C1—O1^iv^	1.2447 (15)
Ca—O1^iii^	2.3644 (12)	C1—C2	1.528 (3)
Ca—N1^iii^	2.6923 (14)	C2—N1^iv^	1.3350 (16)
Ca—N1	2.6923 (13)	C3—C4	1.377 (2)
Ca—N1^ii^	2.6923 (13)	C3—H3	0.9300
Ca—N1^i^	2.6923 (14)	C4—C3^iv^	1.377 (2)
N1—C2	1.3350 (16)	C4—H4	0.9300
			
O1^i^—Ca—O1^ii^	99.72 (2)	O1^ii^—Ca—N1^i^	74.795 (18)
O1^i^—Ca—O1	99.72 (2)	O1—Ca—N1^i^	74.795 (18)
O1^ii^—Ca—O1	131.49 (5)	O1^iii^—Ca—N1^i^	164.58 (4)
O1^i^—Ca—O1^iii^	131.49 (5)	N1^iii^—Ca—N1^i^	100.65 (6)
O1^ii^—Ca—O1^iii^	99.72 (2)	N1—Ca—N1^i^	114.05 (3)
O1—Ca—O1^iii^	99.72 (2)	N1^ii^—Ca—N1^i^	114.05 (3)
O1^i^—Ca—N1^iii^	164.58 (4)	C2—N1—C3	116.03 (15)
O1^ii^—Ca—N1^iii^	74.795 (18)	C2—N1—Ca	113.69 (10)
O1—Ca—N1^iii^	74.795 (18)	C3—N1—Ca	130.28 (11)
O1^iii^—Ca—N1^iii^	63.93 (4)	C1—O1—Ca	128.83 (11)
O1^i^—Ca—N1	74.796 (18)	O1—C1—O1^iv^	126.2 (2)
O1^ii^—Ca—N1	164.58 (4)	O1—C1—C2	116.91 (10)
O1—Ca—N1	63.93 (4)	O1^iv^—C1—C2	116.91 (10)
O1^iii^—Ca—N1	74.796 (18)	N1^iv^—C2—N1	126.74 (19)
N1^iii^—Ca—N1	114.05 (3)	N1^iv^—C2—C1	116.63 (10)
O1^i^—Ca—N1^ii^	74.796 (18)	N1—C2—C1	116.63 (10)
O1^ii^—Ca—N1^ii^	63.93 (4)	N1—C3—C4	121.66 (16)
O1—Ca—N1^ii^	164.58 (4)	N1—C3—H3	119.2
O1^iii^—Ca—N1^ii^	74.796 (18)	C4—C3—H3	119.2
N1^iii^—Ca—N1^ii^	114.05 (3)	C3—C4—C3^iv^	117.9 (2)
N1—Ca—N1^ii^	100.65 (5)	C3—C4—H4	121.1
O1^i^—Ca—N1^i^	63.93 (4)	C3^iv^—C4—H4	121.1

Symmetry codes: (i) *y*−1/4, *x*+1/4, −*z*+3/4; (ii) −*x*+1, −*y*+3/2, *z*; (iii) −*y*+5/4, *x*+1/4, −*z*+3/4; (iv) −*x*+1, −*y*+1/2, *z*.

### Hydrogen-bond geometry (Å, °)

**Table:**

*D*—H···*A*	*D*—H	H···*A*	*D*···*A*	*D*—H···*A*
C3—H3···O1^v^	0.93	2.57	3.3689 (19)	144

Symmetry codes: (v) *y*+1/4, −*x*+5/4, *z*−1/4.
